# Microbial succession in decomposing carrion is driven by time, modulated by insects and microbial perturbation

**DOI:** 10.3389/fmicb.2026.1773208

**Published:** 2026-05-04

**Authors:** Donna B. McIntyre, Philip S. Barton, Sarah Preston, Benjamin M. Long

**Affiliations:** 1 Future Regions Research Centre, Federation University, Mount Helen, VIC, Australia; 2 Graduate Research School, Federation University, Mount Helen, VIC, Australia; 3 School of Life and Environmental Sciences, Deakin University, Geelong, VIC, Australia

**Keywords:** carrion decomposition, insect-exclusion, microbes, microbial perturbation, post-mortem interval

## Abstract

Recent research into decomposition has shed light on the intricate interplay within multi-kingdom communities, with microbial populations emerging as key players in the breakdown process and emphasizing their interactions within the broader decomposer ecosystem. Despite this, the specific roles and regulatory mechanisms of microbial communities remain underexplored. Gaining deeper insight into these dynamics is essential for advancing ecological and forensic sciences. This study examines the role of microbes in decomposition, particularly in relation to insect activity and external microbiome alterations. We used 12 piglet (*Sus scrofa*) cadavers placed in a rural area in southern Australia, to investigate how experimental insect exclusion and microbial perturbation affects decomposition. Bacterial microbial composition was quantified using 16S technology and compared among treatments and time points. Results showed significant shifts in bacterial diversity and abundance across time points sampled, with early-stage decomposition characterized by a higher abundance of Firmicutes and Actinobacteria, followed by a dominance of Proteobacteria in later stages. Principal coordinate analysis confirmed these patterns, showing that while decomposition stage is the primary driver of diversity, insect access and microbial perturbation shape microbial stability and community shifts rather than overall diversity. This challenges assumptions that external factors strongly influence diversity and highlights the need to consider both time and microbial dynamics in forensic applications, particularly for post-mortem interval estimation.

## 1 Introduction

Forensic microbiology has evolved from early bacterial observations a critical tool in forensic science, particularly in post-mortem investigations where the study of microbial communities has provided valuable forensic insights, linking individuals to locations, causes of death, and time since death (Metcalf et al., 2013; Pechal et al., 2013). Advancements in sequencing technologies and bioinformatics have further transformed forensic microbiology by enabling detailed microbial community profiling, leading to the emergence of the necrobiome framework—a model conceptualizing microbial succession on decomposing remains as a potential tool for forensic applications (Benbow et al., 2019; Metcalf et al., 2016; Guo et al., 2016).

Microbial communities play a crucial role in decomposition by breaking down organic matter through enzymatic and biochemical processes (Benbow et al., 2015). Decomposer bacteria facilitate tissue degradation, producing volatile organic compounds (VOCs) that attract insects, further accelerating decay (Benbow et al., 2015; Burcham et al., 2019). This microbial activity follows a predictable trajectory despite environmental fluctuations such as temperature, oxygen availability, and moisture (Cláudia-Ferreira et al., 2023). However, while microbial succession is well-documented, external factors such as microbial perturbation—defined as disruptions to the normal composition or function of microbial communities caused by chemical, biological or environmental influences (e.g., antibiotic use, dietary influences) and insect activity remain poorly understood in forensic contexts. Microbial perturbations have been shown to alter microbial dynamics in other settings, including human health, agriculture, and environmental ecosystems, suggesting their potential to significantly influence decomposition processes.

One major gap in forensic microbiology is understanding how microbiome perturbation before death affects post-mortem microbial succession. The gut microbiome, which plays a central role in decomposition, is highly responsive to pharmaceutical exposure (Chae and Cho, 2020; Benedetti et al., 2019). Antibiotics, in particular, disrupt microbial ecosystems by altering species composition and reducing bacterial diversity (Weersma et al., 2020; Wilson and Nicholson, 2017). While this effect is well-documented in living individuals, few studies have examined how ante-mortem microbiome disruptions influence decomposition (Cláudia-Ferreira et al., 2023). This is crucial as disruptions in the microbial succession could impact key forensic estimations such as post-mortem interval PMI, cause of death determinations, and toxicological interpretations.

In addition to microbial perturbation, insect activity also influences decomposition dynamics (Amendt et al., 2011). Blow flies (Diptera: Calliphoridae) are among the first insects to colonize remains, with their larvae feeding on soft tissues and facilitating bacterial dispersal (Benbow et al., 2019). However, microbial-insect interactions are complex, with evidence suggesting that insects may introduce, suppress, or selectively promote bacterial taxa (Dash and Das, 2020; Pechal et al., 2014). While it is known that insect exclusion slows decomposition (Matuszewski and Madra-Bielewicz, 2019), its effects on microbial succession remain unclear. Some studies suggest that insect activity enhances microbial diversity by promoting bacterial dispersal, while others indicate that insect larvae outcompete certain microbial taxa for resources (Metcalf et al., 2013; Pechal et al., 2014). This interplay highlights the need for controlled experiments that manipulate both microbial perturbation and insect access to assess their combined effects on microbial succession.

In addition to microbial and entomological measures, decomposition progression can be quantified using the Total Body Score (TBS) system, which assigns numerical scores to morphological changes across body regions and provides a standardized, non-destructive method for tracking decay over time (Megyesi et al., 2005). TBS has been widely applied in forensic contexts as an indicator of PMI and in this study it served as a complementary measure alongside microbial community analyses, to guide sampling time points.

Despite growing interest in forensic microbiology, few experimental studies directly manipulate microbial and entomological variables to examine their influence on decomposition (McIntyre et al., 2024). Most studies passively observe microbial succession under natural conditions, making it difficult to quantify and measure the specific contributions of microbial perturbation and insect activity (Cláudia-Ferreira et al., 2023; Li et al., 2016). While reviews acknowledge the potential forensic implications of microbiome disturbances, there remains a lack of empirical data validating these claims. This study aims to address this gap by experimentally investigating bacterial responses to microbiome perturbation and insect exclusion during decomposition stages examined.

This study is guided by two key questions:

1. How do insect access and microbial perturbation influence microbial species richness (alpha diversity) during the examined decomposition stages?
2. How do insect access and microbial perturbation influence microbial species composition (beta diversity and abundance) throughout the examined stages of decomposition?

In this study, we aim to address these questions by manipulating two key variables: microbial perturbation and insect access. We hypothesize that perturbing the microbial environment and excluding insects will influence the microbial species richness (alpha diversity) and composition (beta diversity and abundance) of microbes across three decomposition stages: fresh, active decay, and dry/skeletonized. This study focused on these stages of decomposition, as they represent biologically and forensically distinct phases characterized by pronounced microbial and entomological activity. Intermediate stages were excluded due to their transitional nature and variability, which can obscure stage-specific microbial patterns and reduce interpretability within the study’s experimental design.

## 2 Methodology

We used a randomized block design of 12 piglets locally sourced [stillborn *Sus scrofa* (Linnaeus, 1758)] in a fresh state from a piggery in rural Victoria and confirmed with staff to have similar time of death. The piglets were grouped into three blocks of four piglets across a three-hectare study area (3 blocks × 4 treatments). Blocks were separated by approximately 200 m, and piglets within blocks separated by approximately 20 m. Carcasses were placed in 4-degree Celsius refrigeration overnight in a sealed container (separated according to their treatment group) until placement in the field the following morning at approximately 0900 h. Carcasses were removed from the refrigerator and allowed to warm to ambient temperature (still in the sealed containers), prior to placement in the field. The field was situated on a rural property in Victoria, Australia (143°48′13″E, 37°36′31″S), and the experiment was conducted during 11th February—4th March 2023. Each piglet weighed approximately 1.27 kg (± 0.35 s.d.).

Three replicates of four piglets were exposed to a different combination of insect and microbial exclusion treatments intended to isolate the effects of variation in insects and microbes, and their effects on the microbial community.

These treatments were:

(i) A negative control with no condition/treatment affecting insects or microbes;
(ii) Insect exclusion but microbes undisturbed
(iii) External microbe perturbation but insects present; and,
(iv) Both insect exclusion and external microbe perturbation.

Insect and microbial treatments, and their combinations, were established as follows designed to isolate drivers of variation and research their effects on decomposition, characterized by volatile organic compounds:

- *Negative control:* Piglets placed into a tub, with holes cut in each side and the bottom of the tub, to allow for ventilation and insect access. Piglets in the control group were unwashed.
- *Insect exclusion:* As in (*i*) and insect mesh (18 × 16 stands per inch) was secured over the opening using glue and reinforced with industrial-grade adhesive tape along the edges to ensure complete coverage and prevent insect entry. An insect mesh sleeve was fitted to the tubs to allow for microbial swab sampling without compromising the environment.
- *Microbial perturbation*: As in (*i*) and piglets were washed in chlorhexidine and left to soak in this solution in a large, covered plastic tub overnight. Prior to placement in the field, piglets had chlorhexidine injected into their oral, ear, nasal and anal orifices (approximately 30 mL total).
- *Insect exclusion + microbial perturbation:* involved both (*ii*) and (*iii*) above.

All piglets were placed into individual tubs. Pictures of the experimental set-up and tub design can be viewed in the supplementary materials in McIntyre et al. (2025).

### 2.1 Microbial sampling

The control piglets were selected as the anchor/reference piglet for when all other piglets were to be sampled, in order to establish a consistent reference framework across treatments. All piglets were sampled upon placement in the field for the “fresh” sampled time point (T1) when TBS was lowest (0). Once the control piglet reached “active decay” as indicated by TBS (9–10), time point 2 (T2) was sampled across all piglets. Finally, once the control piglets reached dry/skeletal remains, as indicated by TBS (27), all piglets were again sampled, for time point 3 (T3). The associated accumulated degree hour (ADH) for each sampling time point is referenced in McIntyre et al. (2025). At each sampling time point, piglets were swabbed across multiple body surfaces and orifices using sterile, single-use swabs to obtain an overall profile of the microbial communities present. A single swab was used per individual and samples were pooled at the individual level rather than analyzed by anatomical site.

### 2.2 Sample processing and sequencing

The bacterial communities for microbial swabs collected were assessed by genetic identification employing high throughput sequencing techniques. Following swabbing of the piglets, all swabs were labelled and transferred to the lab on ice. Swabs were stored at –80 degrees Celsius until ready for sending to Australian Genome Sequencing Facility for DNA extraction and 16-S Amplicon sequencing using the conditions shown in Table 1.

**Table 1:** Conditions for DNA extraction and 16-S Amplicon sequencing.

Target	Cycle	Initial	Disassociate	Anneal	Extension	Finish
16S: V3-V4	30	98 °C, 30 s	98 °C, 10 s	60 °C, 10 s	72 °C, 30 s	72 °C, 300 s

DNA was extracted from the swabs using the DNeasy Power Soil Pro Kit from Qiagen (Australia) following manufacturer’s instructions. Negative controls that were included in the extraction process did not show evidence of amplification following gel electrophoresis and were thus not included in sequencing. The negative controls consisted of unused sterile swabs processed alongside samples to monitor for potential contamination. 16S rRNA gene sequencing was performed using Illumina MiSeq with barcoded primers targeting the V3-V4 region: 341F (5′-CCTAYGGGRBGCASCAG-3′) and 806R (5′-GGACTACNNGGGTATCTAAT-3′). Full sequencing data is available at^1^.

### 2.3 Data cleaning and analysis

All analyses were conducted using the R base package version 2023.12.0 + 369 (R Core Team, A and Team, R. C, 2022).

The raw count data was first downloaded from AGRF and the program cutadapt (v4.4) (Martin, 2011) was used to remove the primer sequences. The R package, dada2 (v1.24.0) (Callahan et al., 2016), was then used to filter and trim the sequences using the following parameters maxEE = (2,2), rm.phix = TRUE, minLen = 190, truncLen = (270,220). Before the merging of paired reads, an error model was created, followed by removal of chimeric sequences. The DECIPHER (v2.24.0) (Wright, 2016) package then used to infer the ASV to the reference library (SILVA_SSU_r138_2019.v2).

Following pre-processing the sequences were analyzed using the Phyloseq package (v1.42.0) (McMurdie and Holmes, 2013). A count file, taxonomy file, and sample information file were imported and formatted consistently. These were integrated into a Phyloseq object using the otu_table, tax_table, and sample_data functions for downstream analysis. A phylogenetic tree was generated using the ape package (v5.8–1) (Paradis et al., 2004), and it was incorporated into the Phyloseq object to facilitate further analysis. Basic summary statistics were obtained for the dataset. After cleaning the data, including removing the “T3” time point sample due to being an outlier, we explored the dataset’s structure, including the number of taxa and samples, as well as sample names and taxonomic ranks.

To assess sequencing depth, rarefaction curves were plotted to check for saturation of microbial sequences. The distribution of sequences per sample was visualized using a histogram, which revealed a similar sequencing depth across samples. Additionally, the total number of sequences per sample was calculated, and any variations in read counts were explored further using summary statistics and plots. This workflow enabled the exploration of microbial sequencing data and ensured that the dataset was ready for downstream analyses.

All replicates within each treatment and time point were sequenced and analyzed individually. Diversity metrics (alpha and beta diversity) were calculated for each replicate, and statistical comparisons were performed at the group level. Replicates were not pooled, except in visualization where group means are displayed for clarity.

To assess alpha diversity, we calculated Shannon diversity species richness. We first plotted the richness of the index across time points, with violin plots and points to visualize the distribution of diversity across samples. The Shannon diversity index was specifically calculated using the diversity function from the vegan package, and additional indices, such as richness and evenness, were computed. Richness was estimated using the estimate function (Scire et al., 2023), while evenness was calculated by dividing the Shannon diversity by the logarithm of species count.

We used ggline from the ggpubr (v0.6.0) package (Kassambara, 2018) to visualize diversity indices across time points and groups, including statistical comparisons of mean differences with significance testing. In the statistical analysis, Kruskal-Wallis and paired Wilcox signed-rank tests were conducted to assess differences in Shannon diversity between treatment groups and time points (T1 and T2). All statistical tests were conducted at an alpha level of 0.05.

To explore the differences in microbial community composition, a Principal Coordinate Analysis (PCoA) was utilized to explore differences in Bray–Curtis dissimilarity (Goodrich et al., 2014). A 95% confidence interval ellipse was added for each time point group to visually depict the spread of samples over the PCoA axes. Additionally, a linear regression line was fitted to the data, allowing for an assessment of trends in community composition across the time points.

To analyze microbial community composition at the phylum level, we aggregated data (detection: 50%, prevalence: 70%) and transformed it into relative abundances. Composition was visualized, sorting samples by group and ASVs by abundance. Differences between treatment groups over time were assessed using ANOSIM, followed by pairwise comparisons.

Total DNA base pair extraction per sample was visualized with a bar plot; abundance was plotted across time points and groups. Normality of the DNA base pair extraction data was assessed using the Shapiro–Wilk test. Since the data did not follow a normal distribution (*p* = 0.0077), non-parametric tests were employed for subsequent analyses. A Wilcoxon signed-rank test was used to compare DNA extraction yields between T1 and T2 time points within the same individuals. No significant difference was observed (*p* = 0.25, *V* = 33). In contrast, a Kruskal-Wallis test, which treats samples as independent, indicated a significant difference (*p* = 0.006, *χ*^2^ = 7.55,). Given the paired design of the study, the Wilcoxon test result is more appropriate and was used for final interpretation.

## 3 Results

A total of 1,009,013 reads were generated from 22 samples, and 2,925 taxa were identified. Phyloseq summary statistics can be observed in Table A1 in the appendix. There were 2 samples from T1, 1 sample from T2, and 11 samples from T3 which failed qualify for further testing due to low PCR output. As such, these samples were excluded from further analysis.

**Figure 1:**
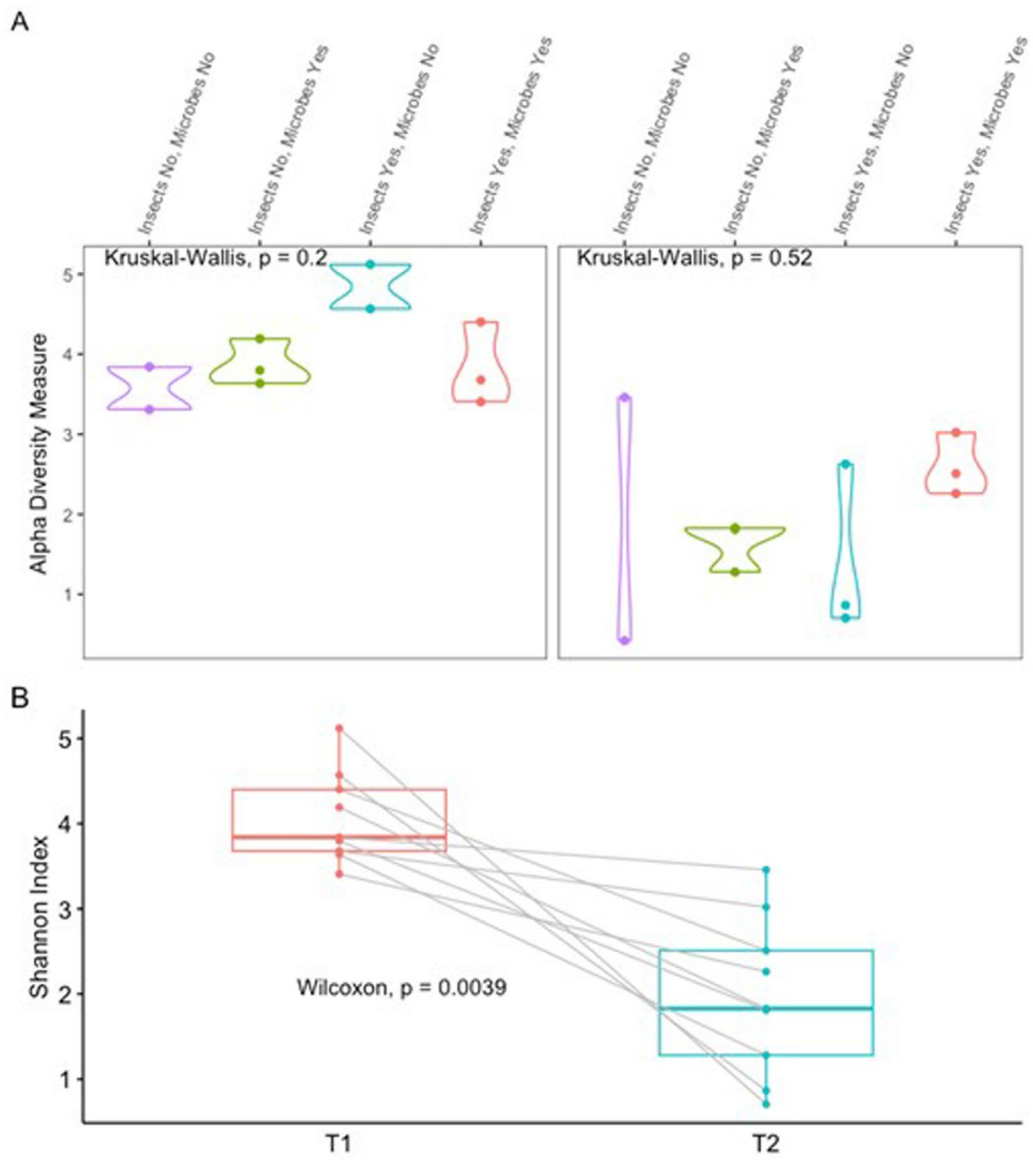
Alpha diversity, as measured by Shannon index. **(A)** Values grouped by time point and four treatment groups: insects no and microbes no (insect exclusion and microbial perturbation), insects no and microbes yes (insect exclusion), insects yes and microbes no (microbial perturbation), and insects yes and microbes yes (control). **(B)** Paired comparisons of Shannon diversity within individual samples between T1 and T2.

Microbial diversity, assessed using the Shannon diversity index, demonstrated significant variation between time points, as can be observed in Figure 1B (Wilcoxin: *p* = 0.0039, *V* = 45). Time point 1 (T1), corresponding to the fresh stage of decomposition had a significantly greater alpha diversity than time point 2 (T2), representing the active stage of decomposition. However, microbial diversity was relatively stable across the experimental groups as there was no significant difference between alpha diversity between the treatment groups at both T1 (Kruskal-Walace: *p* = 0.197, chi-squared = 4.673, *df* = 3) and T2 (Kruskal-Walace: *p* = 0.524, chi-squared = 2.242, *df* = 3).

**Figure 2:**
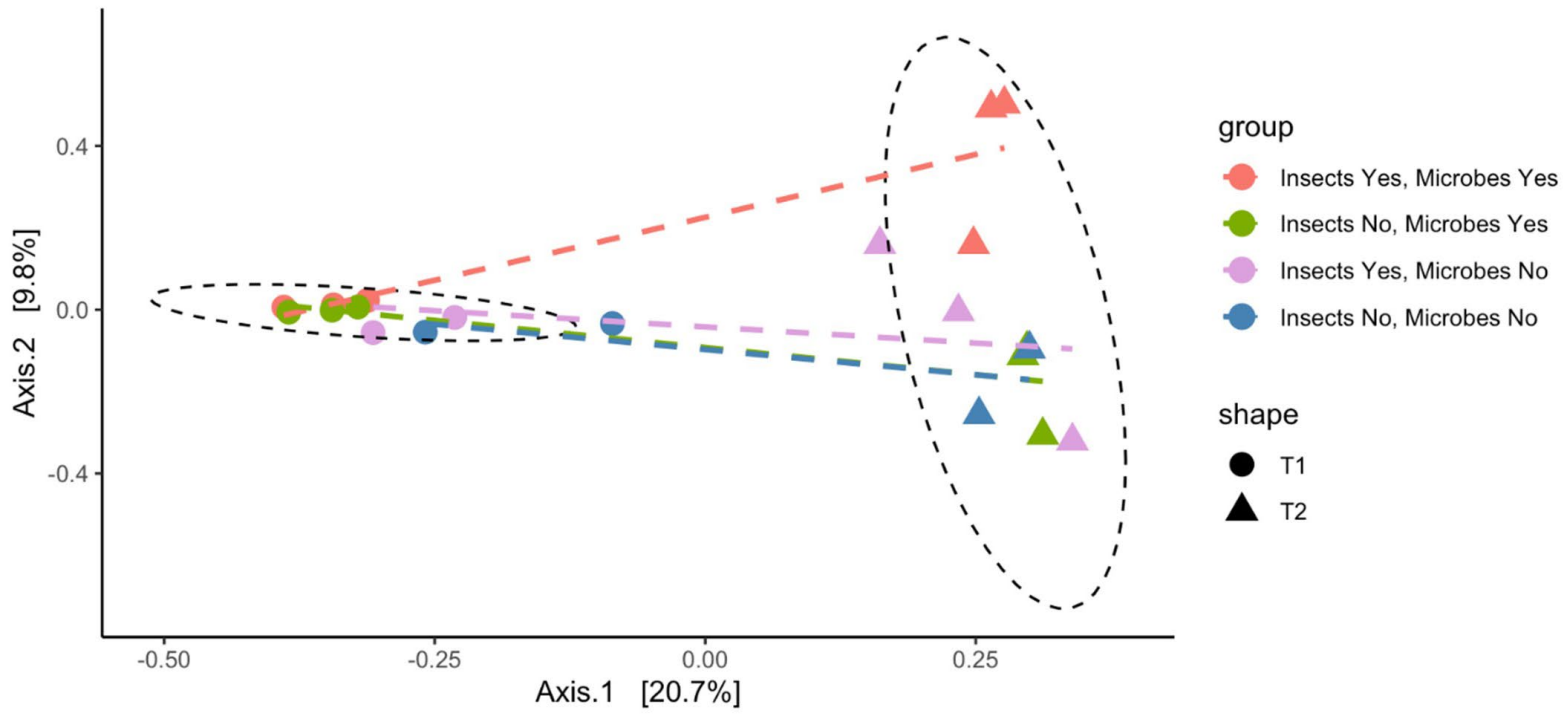
Principal coordinate analysis (PCoA) microbial community dissimilarities between four treatment groups (control = control, Insect_Ex = insect exclusion, Microbe_Ex = microbial perturbation, and Microbe_Insect_Ex = insect exclusion and microbial perturbation) across two sampling time points (T1 and T2). A 95% confidence interval ellipse depicts the spread of samples over the PCoA axes. Dashed lines displays trends in community composition across the time points.

The PCoA plot shown in Figure 2 illustrates the microbial community composition across four treatment groups at two sampling time points (T1 and T2). Dashed lines indicate directional trends in community dissimilarity between the treatment groups over time. At T1, the initial sampling point, the microbial communities were more similar, particularly in samples with an intact microbiome. In contrast, pigs with a perturbed microbiome exhibited the greatest dissimilarity at T1, although this difference was not statistically significant (Kruskal-Wallace*: p* = 0.197, chi-squared = 4.67, *df* = 3). As the experiment progressed, a distinct shift in microbial communities was observed by T2. Despite increased dissimilarities between treatment groups at T2, this difference did not reach a statistical significance. Notably, while the interaction between time point and treatment group significantly influenced the microbial composition, as assessed by distances (ANOSIM*: p* = 0.001, *F* = 2.131, *df* = 7), no significant differences were found between the treatment groups themselves (ANOSIM*: p* = 0.09, *F* = 1.05, *df* = 3).

Building on the overall community composition patterns, phylum-level shifts were observed across time points and treatment groups (Figure 1). At T1, Firmicutes and Actinobacteria were the predominant phyla across all samples, with Actinobacteria particularly enriched in groups with an intact microbiome. In contrast, microbially perturbed groups exhibited a higher relative abundance of Proteobacteria. By T2, Proteobacteria had become the dominant phylum, while Firmicutes remained abundant in over 50% of insect-exposed samples. Notably, Bacteroidota was detected in 80% of T2 samples from insect-excluded groups, but only in 20% of insect-exposed samples. These results indicate a clear shift from Firmicutes and Actinobacteria dominance at T1 to Proteobacteria dominance at T2, with treatment-specific differences influencing phylum-level profiles.

**Figure 3:**
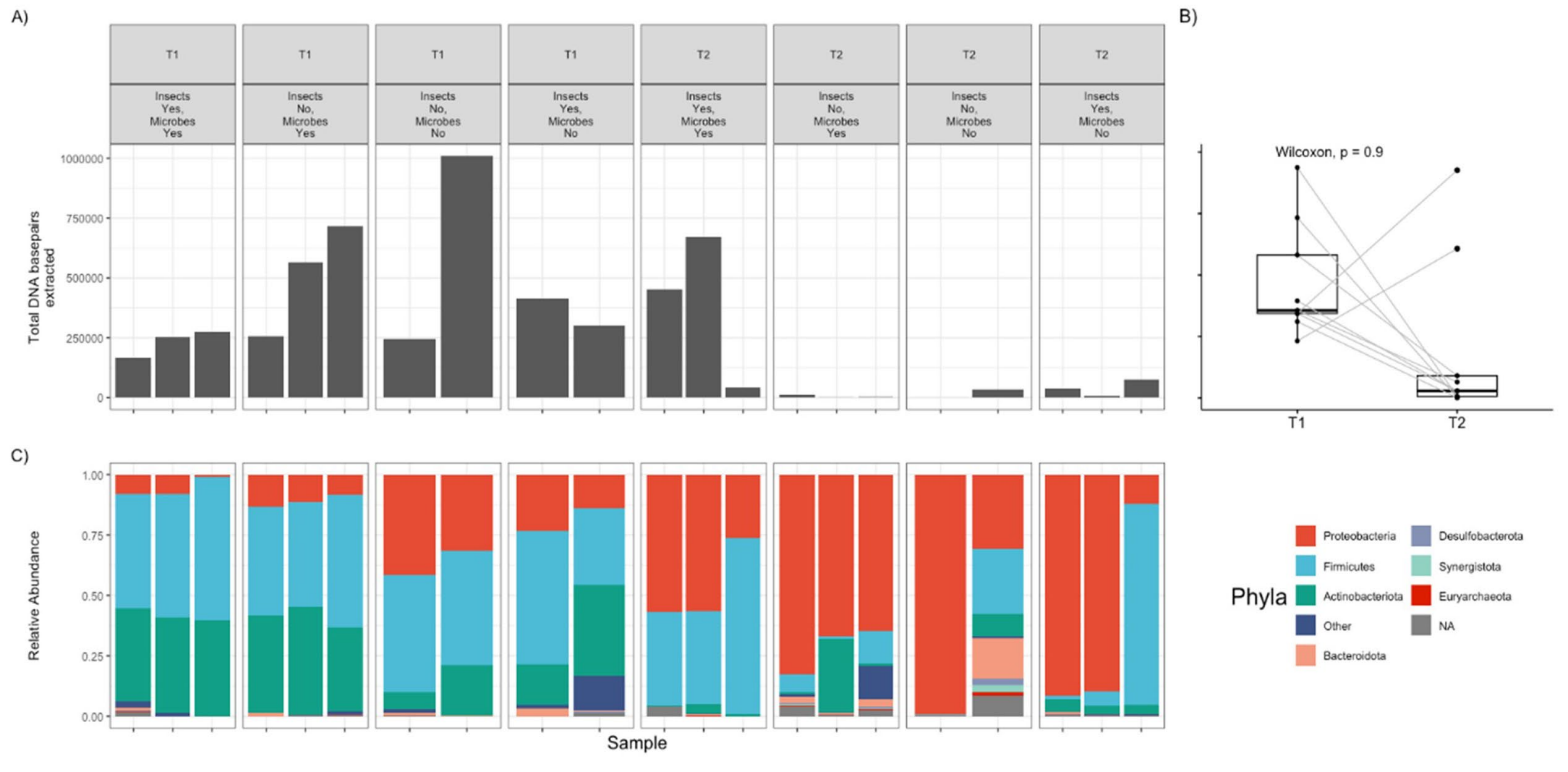
Plot **(A)** illustrates the total DNA base pairs extracted from each sample. Plot **(B)** displays overall paired comparisons of total DNA base pairs extracted within individual samples between T1 and T2. Plot **(C)** demonstrates relative abundance of microbe phyla across T1 and T2 with associated DNA base pairs extracted from each sample, respectively. NA references sequences that were unable to be matched to any known microbe.

In addition to changes in dominant phyla within the microbial communities, total DNA abundance was assessed using base pair read counts. As shown in Figure 3, DNA extraction yields (total base pair reads) were generally higher at T1 compared to T2 across all samples; however, this difference was not statistically significant (Wilcoxin: *p* = 0.90, *V* = 33). At T1, there were no significant differences in DNA yield between treatment groups. In contrast, by T2, a significant difference emerged between the control group and both insect-exclusion treatments (Dunn’s test: *p* < 0.03, chi-squared = 6.379, *df* = 3), while no significant difference was observed between the control and microbial perturbation treatment.

## 4 Discussion

This study represents an exploratory investigation into the combined effects of insect exclusion and microbial perturbation on the post-mortem microbiome. While previous research has typically examined these factors independently, our approach provides preliminary insight into how multiple biotic disturbances may interact to shape microbial succession. The results indicate that decomposition stage is the primary driver of microbial community structure, with treatment-related effects influencing compositional patterns rather than producing consistent shifts in overall diversity. Although the perturbation did not generate strong or sustained changes in alpha diversity, observed compositional differences—such as variation in the relative abundance of specific taxa under insect exclusion—suggest that external ecological factors may modulate community trajectories during early decomposition. Given the limited sample size, and focus on selected decomposition stages, these findings should be interpreted as proof-of-concept evidence supporting the need for more comprehensive, temporally resolved investigations. By integrating microbiome dynamics with insect activity, our research contributes a novel framework for understanding necrobiome interactions and their implications for PMI estimation in forensic science.

### 4.1 Microbial diversity and succession

The results of this study indicate that microbial alpha diversity, as measured by the Shannon diversity index, did not significantly differ between treatment groups, despite variations in microbial perturbation and insect access. Importantly, the bacterial taxa observed in this study exhibited stage-associated patterns consistent with known decomposition processes, providing biological context for shifts in community composition across fresh, active decay, and dry/skeletonised stages. However, there was a significant difference in alpha diversity between the fresh (T1) and active decay (T2) stages of decomposition, suggesting a shift in community complexity over time. This aligns with the well-documented concept of microbial succession during decomposition, where the microbial community evolves over time in response to environmental changes (Benedetti et al., 2019; Guo et al., 2016; Metcalf et al., 2013; Wang et al., 2022).

Despite external perturbations, the microbial community maintained overall diversity, likely due to functional redundancy, where different taxa perform similar ecological roles (Delgado-Baquerizo et al., 2016). However, microbial composition did shift as decomposition progressed. At T1, Firmicutes and Actinobacteria dominated, with Proteobacteria more abundant in microbially perturbed groups. By T2, Proteobacteria had become dominant, particularly in insect-access samples, a pattern aligning with prior decomposition studies (Iancu et al., 2018a; Iancu et al., 2018b). The reduction in DNA yield at T2 further suggests that microbial activity was highest in early decomposition, likely due to the presence of host-associated bacteria, which declined as environmental conditions became more selective.

The lack of significant differences in diversity across treatment groups suggests that, although perturbations such as insect exclusion or microbial disturbance alter microbial composition, they do not substantially affect overall microbial diversity at these time points. This stability in diversity may reflect the influence of broader ecological drivers—such as nutrient availability or environmental conditions—on microbial succession (Delgado-Baquerizo et al., 2016; Allison et al., 2013). The consistent diversity observed in insect-excluded groups, alongside the decline in species richness at T2 in groups with both insect access and microbial perturbation, indicates that microbial communities undergo a robust and structured succession even in the face of external disturbances.

While DNA base pair yields were generally higher at T1 than at T2 across all treatments, this difference was not statistically significant. This suggests a potential overall decline in DNA recoverability over time, possibly due to degradation processes associated with advancing decomposition (Rahi et al., 2021). However, the absence of statistical significance may reflect high variability within the data or insufficient statistical power to detect subtle differences, potentially due to limited sample size. Additionally, meaningful changes in less abundant microbial taxa may have been obscured by the dominant presence of more abundant species.

Interestingly, although there were no significant differences in base pair extractions between treatment groups at T1, clear distinctions emerged by T2. Specifically, the control group exhibited significantly higher DNA yields than both insect-exclusion treatments. This finding highlights the potential role of insect activity in enhancing microbial DNA, perhaps through facilitation of microbial dispersal (Gurung et al., 2019; LeBlanc et al., 2019). In contrast, microbial perturbation alone did not significantly alter DNA yields compared to the control, suggesting that insect presence may exert a stronger influence on microbial community structure or biomass than microbial disturbance in isolation. A notable observation was the high DNA extraction yield in a microbially perturbed pig at T1, potentially due to chlorhexidine’s disruption of bacterial membranes (Milani et al., 2021), temporarily increasing free-floating DNA. There is limited research on the degradation of microbial DNA in decomposition investigations however, we hypothesize that DNA degradation by T2 may be due to enzymatic degradation by microbial nucleases (Dash and Das, 2018), environmental exposure (e.g., UV radiation, temperature fluctuations, and moisture) (Alaeddini et al., 2010; Cossette et al., 2021; Janaway et al., 2009; Rahi et al., 2021), and the increasing dominance of decomposer taxa which were not sequenced (such as fungi) that rapidly degrade available bacterial genetic material (Apere, 2024; Vara, 2017).

### 4.2 Insect-microbial interactions

Although insect taxa were not characterised within the scope of the present study, the insect community associated with this experimental system has been described previously (McIntyre et al., 2024), and insect presence versus exclusion provides important biological context for interpreting microbial differences observed between treatments. Insects play a crucial role in decomposition, influencing microbial dispersal and activity. Blow flies and beetles introduce bacteria through feeding and colonization (Gurung et al., 2019; LeBlanc et al., 2019). Differences between insect-access and insect-excluded groups (such as the prevalence of Bacteroidota in insect exclusion treatments) suggest that insect digestion and metabolic byproducts alter the carrion microbiome. Additionally, insects release scent signatures (Frederickx et al., 2012), contributing to volatilome variation, which in turn influences microbial community structure. Insects also modify the microbial landscape through competitive interactions, influencing succession patterns (Burkepile et al., 2006). This interaction is dynamic; as insects modify the microbial environment, the changing VOC profile can, in turn, attract or repel other insect species, creating a feedback loop that influences the decomposition process (Zheng et al., 2013; Burkepile et al., 2006).

### 4.3 Microbial community composition and forensic relevance

Microbial beta diversity showed increased divergence between treatment groups over time. While microbial communities were initially similar across samples, differences became more pronounced at T2. However, statistical significance was not reached, possibly due to sample size constraints and overlapping microbial shifts across treatments. This suggests that microbial succession follows predictable patterns despite initial perturbations.

The most abundant phyla across all samples were Proteobacteria, Firmicutes, Bacteroidota, and Actinobacteria—taxa commonly associated with decomposition (Chun et al., 2015). Proteobacteria, particularly Pseudomonas and Enterobacter, are key players in organic matter degradation and VOC production (Dash and Das, 2018). Their increasing abundance over time, alongside Firmicutes and Bacteroidota declines, aligns with known decomposition trajectories (Li et al., 2024). Interestingly, Bacteroidota was prevalent in insect-excluded samples, suggesting that insect activity alters oxygen availability, favouring certain microbial groups.

Actinobacteria were most prevalent early in decomposition, reflecting their role in organic matter breakdown (Hilal et al., 2022). It is important to consider that environmental and host factors can influence microbial patterns. Singh et al. (2018) showed that Actinobacteria respond to accumulated precipitation, and Gammaproteobacteria to the decedent’s BMI, which may help explain some of the variation observed here. Tarone et al. (2022) demonstrated that insect presence, swab location, and thermal conditions can shape microbial communities, suggesting that differences between insect-access and insect-excluded groups may reflect more than the experimental treatments alone. Additionally, Matuszewski et al. (2014) and Dawson et al. (2020) highlighted that carcass size and diet affects insect colonization patterns, which likely influences microbial succession indirectly. Accounting for these factors provides broader ecological context for the results and aids in meaningful comparisons with previous studies.

### 4.4 Forensic implications

Our study highlights the complexity of microbial succession in decomposition and its forensic relevance. While microbial perturbations and insect exclusion altered community composition, their effects were not always statistically significant, suggesting a degree of microbial resilience. This has important implications for forensic investigations, as it indicates that decomposition-associated microbial communities may follow predictable patterns despite environmental disturbances.

Key microbial taxa identified—Proteobacteria, Firmicutes, Bacteroidota, and Actinobacteria—play fundamental roles in decomposition and VOC production, which are critical for PMI estimation (Metcalf et al., 2013). The observed microbial shifts, particularly the rise of Proteobacteria and decline of Firmicutes, suggest that incorporating microbial data into PMI models could enhance forensic accuracy. The finding that Bacteroidota proliferated in insect-excluded environments further highlights the need to consider ecological interactions in forensic applications.

These findings highlight the complexity of microbial community dynamics during decomposition and suggest that while microbial perturbations and insect exclusion can influence succession patterns, their long-term effects may be moderated by other ecological processes. Microbial analysis is still a developing field in forensic science, but these findings suggest its potential for court cases. If microbial diversity can be reliably tied to decomposition stages, it could be used to back up PMI estimates in a legal context, especially in cases where the timing of death is crucial to the investigation. Experts may need to communicate the complexity of microbial patterns to legal professionals in a way that helps them understand how microbial signatures can be interpreted in the context of forensic evidence.

### 4.5 Future research considerations

A key limitation of this study is the extent to which microbial perturbation was successfully achieved. While chlorhexidine application was designed to disrupt the initial microbial community, the similarity in microbial community composition between perturbed and control groups suggests that this intervention may not have been sufficient to produce a strong or lasting shift in the microbiome. It is possible that microbial recolonization occurred rapidly after treatment, or that the perturbation primarily affected external rather than internal microbes, limiting its overall impact on decomposition dynamics. Future studies could address this by manipulating the microbiome ante-mortem (e.g., through administration of antibiotics or dietary interventions) or by applying repeated or alternative post-mortem perturbations. Such approaches may provide a more representative model of how pharmaceutical, or lifestyle factors influence the necrobiome and, in turn, decomposition processes relevant to forensic applications. An additional limitation of this study is the exclusion of intermediate decomposition stages, such as bloat. These stages represent transitional periods characterized by overlapping microbial and entomological processes, which can introduce substantial variability and obscure stage-specific microbial signatures. The study therefore focused on fresh, active decay, and dry/skeletonized stages, which represent biologically distinct phases of decomposition and allowed for clearer interpretation of microbial succession patterns within the scope of the experimental design. Further research incorporating more granular temporal sampling and diverse environmental variables will be crucial in refining these forensic applications and enhancing our understanding of microbial community dynamics in post-mortem investigations. Notably, none of the T3 samples were included in the analysis due to insufficient DNA yields; this highlights a key limitation in the current approach and illustrates the need for more sensitive DNA extraction and amplification methods particularly in the late-stage decomposition in future research. Investigating microbial communities and insect activity across different geographic regions will help determine how local factors influence decomposition and post-mortem interval estimations. Furthermore, studies incorporating site-specific sampling across anatomical regions would provide greater spatial resolution of post-mortem microbial succession. While pooling samples enabled an integrated assessment of the carrion-associated microbiome, anatomical sites are known to harbor distinct microbial communities during decomposition. Analyzing these regions independently would strengthen cross-stage comparisons and refine forensic interpretations. Additionally, field-based studies in uncontrolled settings could provide a more comprehensive understanding of decomposition processes in real-world scenarios. Building on region-specific insights, future studies can refine forensic methodologies by integrating broader ecological and environmental influences, enhancing the applicability of microbial and entomological data in forensic science. Given the challenges in identifying distinct microbial signatures specific to the PMI, an important next step is to investigate bacterial species or strains that could consistently indicate the timing of decomposition. We acknowledge that chlorhexidine, as a broad-spectrum antiseptic, may produce different outcomes compared to antibiotics, which target specific bacterial taxa. However, we contend that the use of chlorhexidine—designed to broadly eliminate microbial populations—offers a more rigorous test of our research question: whether alpha and beta diversity remain stable following microbial perturbation. As such, these findings may be more robust and broadly applicable due to the comprehensive nature of this perturbation approach.

## 5 Conclusion

This study provides a comprehensive exploration of the role of microbial communities and insect activity in the decomposition process, offering valuable implications for forensic science, particularly in the estimation of post-mortem interval (PMI). Our findings demonstrate that microbial community composition evolves over time, with significant shifts occurring as decomposition progresses. While insect activity supports a more diverse and stable microbial environment, its exclusion results in reduced microbial diversity, especially during the active decomposition phase. Similarly, perturbing the microbiome disrupts community dynamics, accentuating the importance of microbial interactions in the decomposition process.

Although treatment effects were not strong enough to produce significant divergence at individual time points, the interaction between time and experimental conditions revealed that microbial succession is influenced by both environmental and perturbative factors. Importantly, despite these perturbations, microbial communities still followed a predictable succession pattern, reinforcing the notion that decomposition-associated microbial shifts are driven by broader ecological and environmental factors. These findings emphasize the complexity of microbial community dynamics during decomposition and suggest that insect exclusion and microbial perturbation can influence but do not entirely determine microbial succession.

## Data Availability

The original contributions presented in the study are included in the article/supplementary material, further inquiries can be directed to the corresponding author.

## Footnotes

1
https://doi.org/10.5281/zenodo.18765807
